# Measuring lip force by oral screens Part 2: The importance of screen design, instruction and suction

**DOI:** 10.1002/cre2.87

**Published:** 2017-10-11

**Authors:** Madeleine Wertsén, Manne Stenberg

**Affiliations:** ^1^ Hospital Dentistry, Special Dental Care Sahlgrenska University Hospital/Mölndal Sweden; ^2^ Dept of Signals and Systems Chalmers University of Technology Sweden

**Keywords:** intraoral pressure, lip force, oral motor dysfunction, oral rehabilitation, oral screen

## Abstract

The aim of this study was to find a reliable method for measuring lip force and to find the most important factors that influence the measurements in terms of magnitude and variability. The hypothesis tested was that suction is involved and thus the instruction and the design of the oral screen are of importance when measuring lip force. This is a methodological study in a healthy population. This study was conducted in a general community. The designs of the screens were soft and hard prefabricated screens and 2 semi‐individually made with a tube allowing air to pass. The screens and the instructions squeeze or suck were tested on 29 healthy adults, one at a time and on 4 occasions. The test order of the screens was randomized. Data were collected during 4 consecutive days, and the procedure was repeated after 1 month. The participants were 29 healthy adult volunteers. The instruction was an important mean to distinguish between squeezing and sucking. The design of the screen affected the lip force so that it increases in relation to the projected area of the screen. A screen design with a tube allowing air to pass made it possible to avoid suction when squeezing. By measuring with and without allowing air to pass, it was possible to distinguish between suction related and not suction related lip force. The additional screen pressure when sucking was related to the ability to produce a negative intraoral pressure. In conclusion lip force increases in relation to the projected area of the screen, sucking generally increases the measured lip force and the additional screen pressure when sucking is related to the ability to produce a negative intraoral pressure.

**Abbreviation**
OSP: oral screen pressure

## 1 INTRODUCTION

Orofacial dysfunction caused by neurological disorders such as stroke, peripheral palsy, operation of brain tumors, infections, head, and neck cancer results in drooling, accidental biting, food retention, and problems to form and transport a bolus. In rehabilitation, prefabricated and individually oral screens are commonly used. Studies of the outcome of the training are unclear as different soft and hard prefabricated oral screens have been used. Thus, there is a great need for reliable, sensitive, and objective methods evaluating orofacial muscle function (Trottman, Phillips, Faraway, & Ritter, 2003).

One of the more common methods to measure muscle strength is to use a handheld dynamometer. It is portable, convenient, and easy to use (Lu et al., 2011). Evaluation of lip force has been carried out with a similar measuring device, LF 100 (Hägg, Olgarsson, & Anniko, 2008; Sjögreen, Lohmander, & Kiliaridis, 2011). Healthy volunteers have been tested in order to investigate lip force, and excellent intrainvestigator reliability was found testing both patients and controls using a hard prefabricated oral screen (Hägg et al., 2008). Lip force and intraindividual variability has been tested on healthy adults, using a soft oral screen, Ulmer large, by testing on two occasions (Sjögreen et al., 2011). The impact of the size of the screen and the lip force executed has been shown to be of great importance (Wertsén & Stenberg, 2017). A small screen scores the smallest value and the largest screen the highest (Table 1).

**Table 1:** Lip force (LF) of healthy controls in previous studies

Type of screen	LF (*N*) + *SD*	*n*	Ref
Ulmer large soft	21 + 7.8	56	Sjögreen et al. (2007)
Ulmer large soft	29 + 9	50	Sjögreen et al. (2011)
OS/II hard prefabricated	24.7 + 6.3	42	Hägg et al. (2008)
Small hard semi‐individual	17.6 + 4.8	24	Wertsén and Stenberg (2017)
Medium hard semi‐individual	21.8 + 5.4	24	Wertsén and Stenberg (2017)
Large hard semi‐individual	32.4 + 7.3	24	Wertsén and Stenberg (2017)

*Note. SD* = standard deviation.

Furthermore, it was shown that by dividing the measured lip force by the screen size, measured as the projected area, the influence of screen size could be eliminated. By measuring oral screen pressure (OSP) in a pressure unit, for example, kPa (kilopascal) measurements from screens with different areas can be compared (Wertsén & Stenberg, 2017). When sucking, apart from the perioral muscles, a great number of muscles are involved. Thus, the measured force will not reflect the force performed by the perioral muscles. In the previous study, the screens were made with a small tube allowing air to pass in to the oral cavity thus preventing the test person to use suction during the measurement (Wertsén & Stenberg, 2017). This option was not available in other studies (Hägg et al., 2008; Sjögreen et al., 2011). Eklund and Eklund who used the screen OS/II in a pilot study also discussed the influence of sucking. They found that some of the test persons had a tendency to suck the screen. This, as well as clenching the jaws, increased the measuring values markedly (Eklund & Eklund, 2007). They also stated that the measurement value was not influenced by different head or seating positions. In other studies, the subjects were instructed to keep the screen as long as possible inside the lips resisting the increasing force for as long as possible (Hägg et al., 2008; Sjögreen et al., 2007). This instruction makes it uncertain whether the subjects have been able to avoid suction or not. However, the studies are few in numbers and have been performed with different kinds of oral screens, hard, soft, and semi‐individually made. Thus, the values reported are difficult to compare.

Changes in intraoral pressure are required to implement swallowing, mastication, or speech. Engelke proposed that several structures operating as biofunctional compartments and valves are participating during swallowing. These collaborate in a coordinated interaction following a dynamic pressure gradient (Engelke, Jung, & Knösel, 2011). Santander showed that intraoral pressure varied significantly depending on different bolus used and its consistency (Santander, Engelke, Olthoff, & Völter, 2013). As a consequence, it is of interest to investigate if it is possible to clearly separate the measurements with an oral screen for squeezing and sucking using different instructions. The aim of this study was to compare soft and hard prefabricated oral screens and two semi‐individually made screens to get separate values for squeezing and suction.

## 2 MATERIALS AND METHODS

The Ethics Committee of the University of Gothenburg approved the study (Dnr. 492‐12), and it was performed in accordance with the Declaration of Helsinki.

### 2.1 Test subjects

Twenty‐nine healthy adults, 20 females and 9 males, aged 31–68 years with ordinary morphology of the face, normal oral motor function, and occlusion were recruited on voluntary basis for the study. They mainly comprised dental health personnel at the Public Dental Service clinic in Mölndal, Sweden. All the individuals gave their informed consent to participate and continued through the entire testing period.

### 2.2 Standardized oral screens

**Figure 1:**
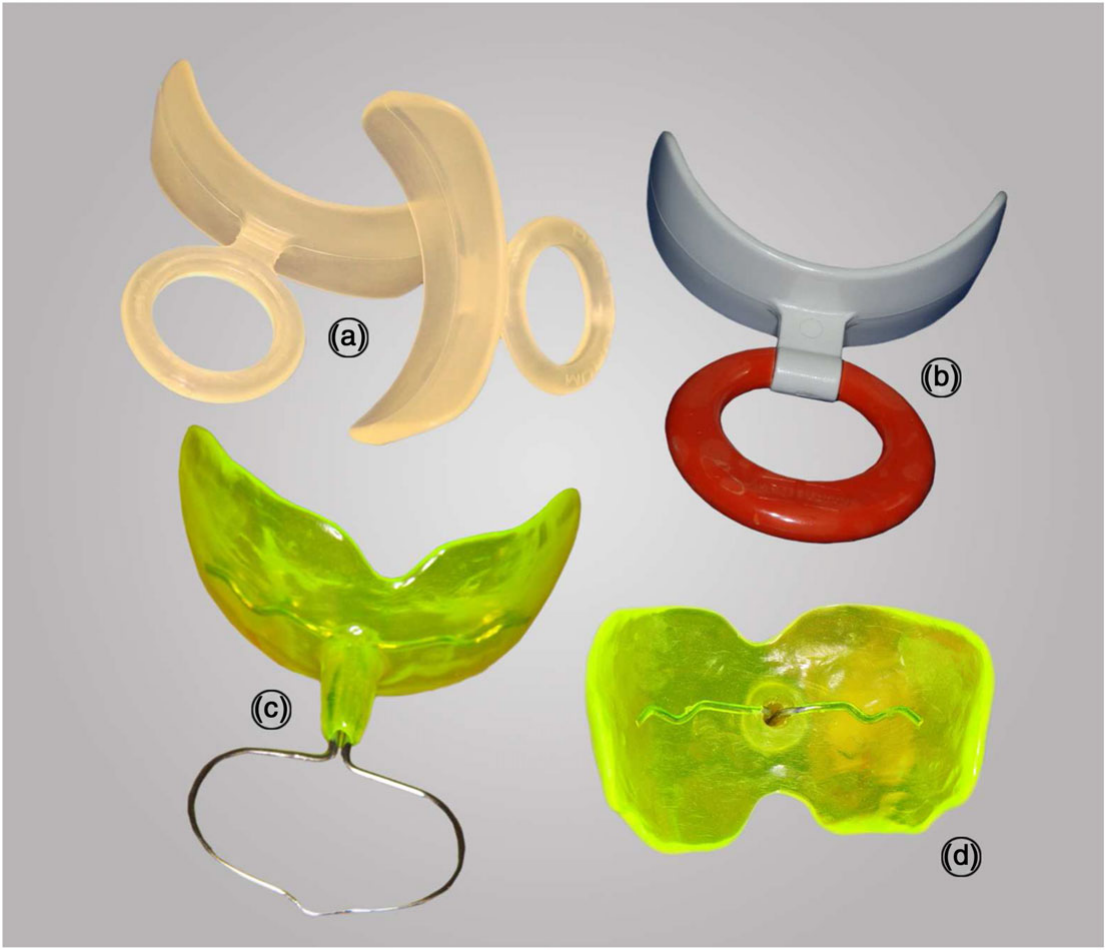
(a) Soft prefabricated oral screen. (b) Hard prefabricated oral screen. (c) Semi‐individual oral screen. (d) Semi‐individual oral screen showing the hole through the central tube

A soft prefabricated screen (Figure 1a), Ulmer large, and a hard prefabricated screen, Oral Screen OS/II (Figure 1b), were ordered from a dental supplier.

Two sizes of oral screens, medium and large, were made in acrylic from plaster casts measuring 49 and 56 mm between the buccal surfaces of teeth 15 and 25. The oral screens were made with a hole and a small tube around the handle (Figure 1c–d). The tube made it possible to let in air, to prevent sucking, or to close it with a piece of wax to enable sucking. The screens were labeled A for the soft prefabricated screen, B for the hard prefabricated screen, C for the medium sized semi‐individual screen, and D for the large sized semi‐individual screen.

### 2.3 Procedure for measuring projected area of the oral screen

The area of the different screens was determined by projecting as in a previous study (Wertsén & Stenberg, 2017). To investigate if the deformation of the soft screen, when pulling, would influence on the projected area, it was fixed with transparent tape in a pellucid tube. The screen was then exposed to the same forces that were measured with the testing subjects.

### 2.4 Lip force measuring

For evaluation of lip force, the lip force meter LF 100 was used (Hägg et al., 2008; Sjögreen et al., 2011).

### 2.5 Measurement procedure

A dental chair with arm and foot rests was used. The examiner started by demonstrating the measuring procedure and gave a verbal instruction. The measuring procedure was carried out with and without suction. Instruction without suction: “Hold the oral screen in your mouth and squeeze your lips as firmly as you can, while I pull it out.” Instruction with suction: “Hold the oral screen in your mouth and suck as hard as you can while I pull it out.”

The test person placed the oral screen in the vestibulum. The wire was stretched in a straight angle, and an assistant started the measuring period of 10 s. The examiner pulled the wire and gradually increased the power until the oral screen was pulled loose. The maximum value was noted. The procedure was repeated three times in a sequence, and all the values were used in the statistical analyses.

### 2.6 Data collection

The order, in which the screens were tested, was randomized. Data were collected on approximately the same hour during four consecutive days. Four oral screens were tested for each of the 29 subjects, one each day. Two instructions were given, and three measurements for each instruction were carried out. Each screen type was tested at two occasions 1 month apart. To squeeze was always the first instruction given at each session. The test persons rested for 3 min between the two instructions. All measured values were registered in Newton. In total, 174 measurements were carried out for each oral screen and instruction. The same investigator made all measurements.

### 2.7 Statistical analysis

The dataset was first analyzed in MS Excel for calculating the OSP by dividing force values with appropriate screen areas. Ninety‐five percent confidence levels of the different means were calculated from *t* statistics with *df* = 173 for the overall means, *df* = 28 for the distribution of individual means, and *df* = 5 for individual means. The sample correlation coefficient (*r* ^*2*^) was calculated in the MS Excel graphs. The individual means data were analyzed in SPSS for normality by the Shapiro–Wilk test. Confidence ellipses for the bivariate Gaussian distribution were calculated from the chi‐square distribution with elliptical axes = 2*kσ* with *k* = 1.177 for 50% confidence and *k* = 2.448 for 95% confidence.

### 2.8 Pilot vacuum experiment

One of the screens (Screen C) was modified to permit measurement of the established vacuum in the oral cavity during lip force measurement. A silicone tube was used to connect the low‐pressure side of a differential pressure transducer (Freescale Semiconductor MPX2050) to the central tube of the screen. The high‐pressure side of the sensor was exposed to the room atmosphere. The sensor output voltage was amplified by an instrumentation amplifier (INA126) and recorded in a personal computer by a multifunction USB device (National Instrument USB‐6008). By using a silicone tube filled with water, the sensor could be calibrated by well‐defined hydrostatic water columns. The sensor sensitivity was found to be 0.109 V/kPa. Estimated uncertainty based on sensor data was a maximum error of less than ±0.2 kPa in the range 0–50 kPa. A small control group of six healthy adults were recruited on a voluntary basis for this test. The measurements were carried out as described previously with three measurements for each of the two instructions at the same (one) occasion. The intraoral negative pressure was recorded during the lip force measurement with suction, and the maximum value of the differential pressure was selected for further analysis.

## 3 RESULTS

The labelling and projected area of the different screens used is presented in Table 2. The area of the soft screen was initially 10.9 cm^2^ and reduced to 8.3 cm^2^ when it was subjected to a load of the same magnitude as the average lip force (18–25 N). The pressure was subsequently calculated with the reduced area. The error in area measurement for all screens is estimated to maximum 5%.

**Table 2:** Overall mean oral screen pressure values for instruction “Squeeze the lips as firmly as you can”

Screen	Projected area (cm^2^)	Mean value (kPa)	Standard deviation (kPa)	95% Confidence interval for mean (kPa)
A	8.3	22.4	6.88	21.3–23.4
B	11.1	23.4	7.63	22.2–24.5
C	15.5	15.5	3.63	15.5–16.0
D	22.6	15.7	3.47	15.2–16.2

*Note*. Screen A = soft prefabricated screen; B = hard prefabricated screen; C = medium sized semi‐individual screen; D = large sized semi‐individual screen.

When evaluated as an OSP, the measurements for the different screens could be compared. In Table 2, an overall picture is presented for the instruction “Squeeze the lips as firmly as you can.” Here, the overall mean value for each screen is based on 174 measurements. We can see that the mean values are grouped in two, one value around 22.9 kPa for Screens A and B and another lower value around 15.6 kPa for Screens C and D. However, with the other instruction “Suck the screen as hard as you can,” there is a different result. The holes in Screens C and D are now sealed with wax allowing suction. In Table 3, we can see that the mean values are grouped in two, but now the Screens A and B are giving the lower group value.

**Table 3:** Overall mean oral screen pressure values for instruction “Suck the screen as hard as you can”

Screen	Projected area (cm^2^)	Mean value (kPa)	Standard deviation (kPa)	95% Confidence interval for mean (kPa)
A	8.3	29.6	7.23	28.5–30.7
B	11.1	30.8	7.53	29.7–31.9
C	15.5	40.2	11.8	38.4–41.9
D	22.6	35.9	8.80	34.6–37.3

**Figure 2:**
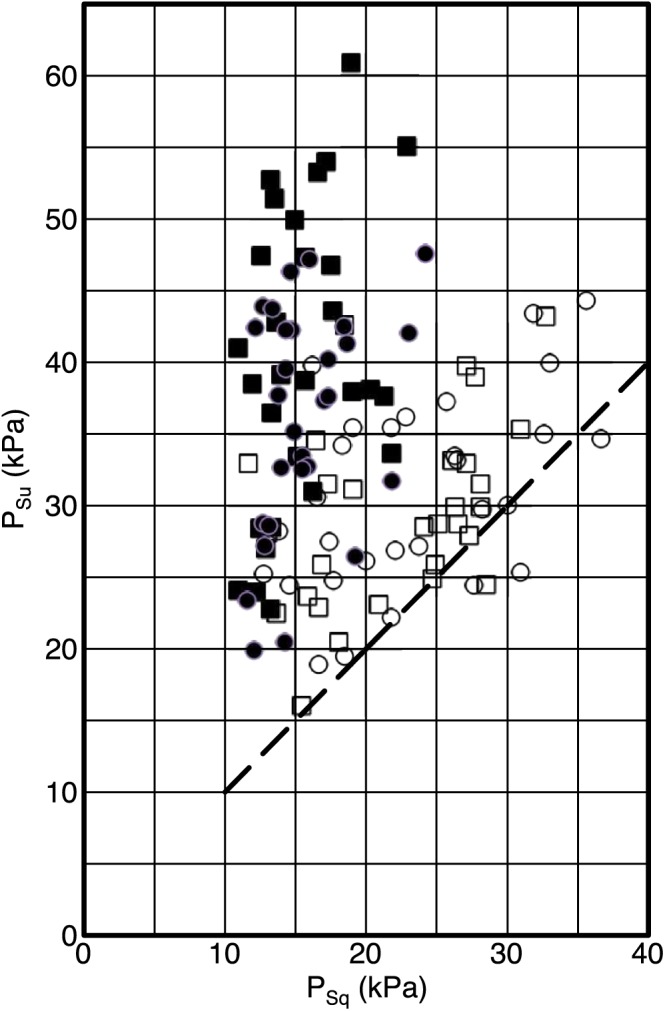
Oral screen pressure measured for individual subjects (*N* = 29) with different instructions, on y‐axis “Suck the screen as hard as you can” (*P*_su_) and on x‐axis “Squeeze the lips as firmly as you can” (*P*_sq_). The different screens were Screen A (□), Screen B (○), Screen C (■), and Screen D (●). Filled markers represent screens with open tube when squeezing. Dashed line indicates equal lip pressure for the two instructions

In Figure 2, the same data set as in Tables 2 and 3 is presented distributed on individual averages. Every individual mean value is based on six measurements distributed on two occasions and three measurements at each occasion. For almost all individuals and screens, the instruction “Suck” resulted in a higher OSP value than “Squeeze.” However, two clusters can be identified. Measurements from Screens A and B are distributed quite close to the line with equal OSP values for the two instructions, whereas measurements from Screens C and D gave significantly higher OSP values when suction was permitted. The results in Figure 2 indicate that when suction is possible as for Screens A and B, there is a possibility that some suction is executed even during the instruction “Squeeze.”

**Figure 3:**
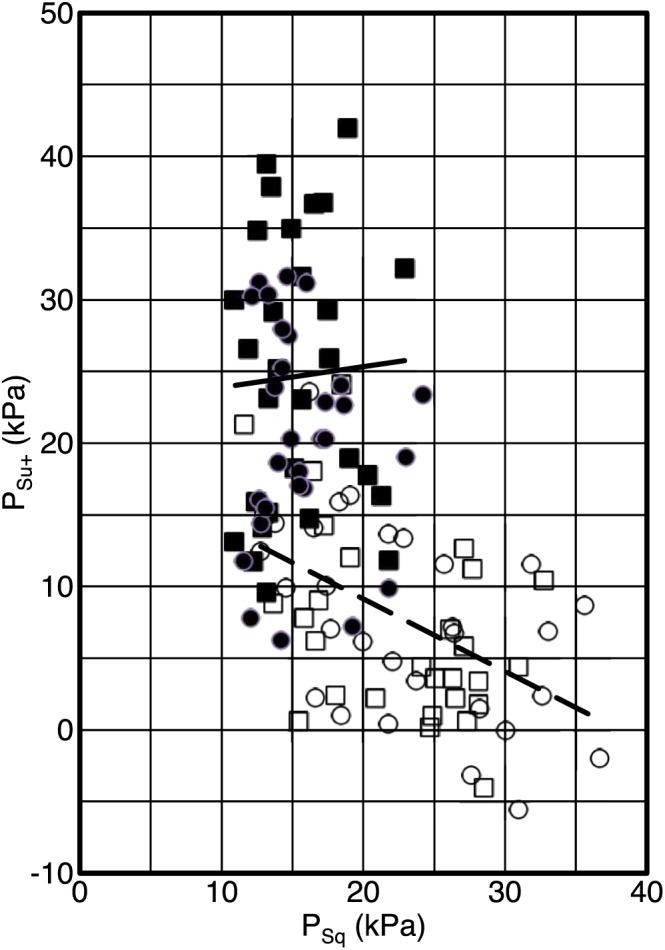
Same data as in Figure 2. The additional oral screen pressure from suction *P*_su+_ is evaluated as the difference between *P*_su_ (oral screen pressure “Suck”) and *P*_sq_ (oral screen pressure “Squeeze”). The different screens were Screen A (□), Screen B (○), Screen C (■), and Screen D (●). Solid line is the trendline for Screen C (*r* ^*2*^ = 0.0024), and dashed line is trendline for Screen B (*r* ^*2*^ = 0.264)

In order to check the influence of suction on OSP measurements, the data were further processed. In Figure 3, the additional OSP from suction *P*_Su+_ is evaluated as the difference between measured OSP at instruction “Suck” *P*_Su_ and OSP measured at instruction “Squeeze” *P*_Sq_.

$$P_{\mathrm{Su}+}=P_{\mathrm{Su}}-P_{\mathrm{Sq}}.$$

The two clusters can still be identified, but there is now a significant difference between screens with and without a hole preventing suction. For Screens C and D, there is almost no correlation between *P*_Su+_ and *P*_Sq_. The trend line for Screen C is almost horizontal with a coefficient of determination *r* ^*2*^ = 0.0024 indicating that the parameters *P*_Su+_ and *P*_Sq_ are practically uncorrelated. On the other hand, with Screens A and B, it is not possible to extract the contribution from suction. The trend line for Screen B has a negative slope with a much higher coefficient of determination of *r* ^*2*^ = 0.264 indicating that suction may be present with the squeeze instruction.

**Figure 4:**
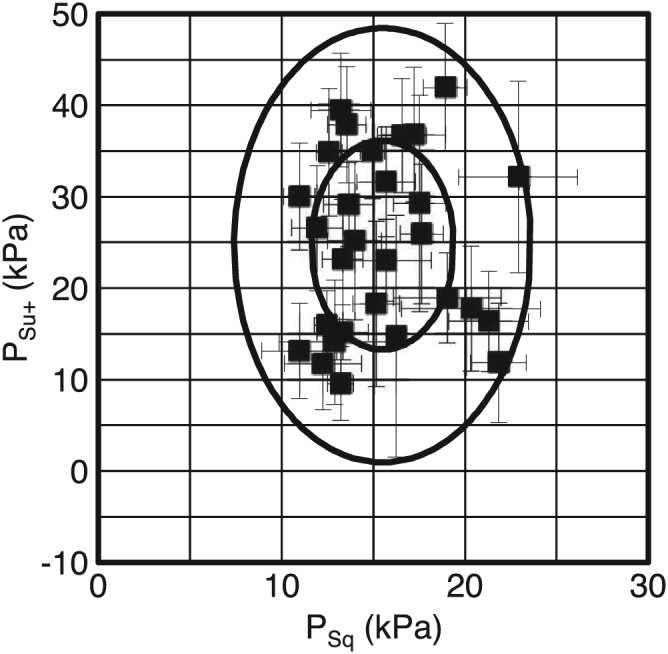
Data as in Figure 3 for Screen C showing the distribution of individual mean oral screen pressure values in the test group around the overall mean. Error bars indicate 95% confidence limits of individual means. Inner ellipse corresponds to mean values 50% confidence ellipse, and outer ellipse corresponds to 95% confidence ellipse

The results from Screens C and D motivate the definition of two independent OSP parameters *P*_Su+_ and *P*_Sq_. These parameters are summarized in Table 4 based on measurements from Screen C. Both parameters may well be normally distributed because the *p* values in the Shapiro–Wilk tests were greater than 0.05. In Figure 4, individual means for the test group is shown together with estimated confidence ellipses around the overall mean. These regions correspond to 50% and 95%, respectively, of a healthy control group.

**Table 4:** Properties of individual oral screen pressure means for the control group (*N* = 29) and Screen C divided into two independent parameters: Oral screen pressure from squeezing (*P*_Sq_) and additional oral screen pressure from sucking (*P*_Su+_)

Parameter	Mean value (kPa)	Standard deviation (kPa)	95% confidence interval (kPa)	Normality (*p* value Shapiro–Wilk)
*P*_Sq_	15.5	3.31	8.77–22.1	0.053
*P*_Su+_	24.7	9.70	5.28–44.1	0.121

**Figure 5:**
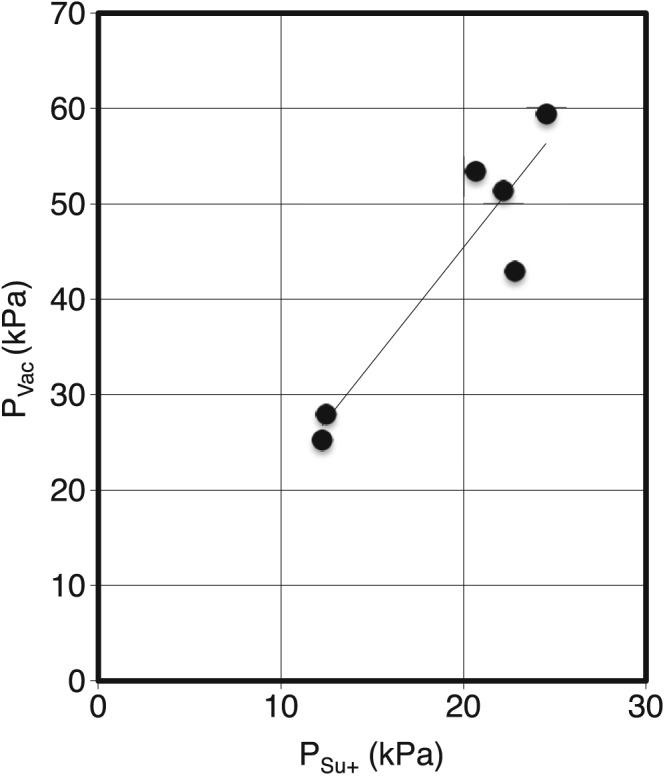
Pilot experiment showing the relation between the negative pressure inside the mouth *P*_Vac_ and the additional oral screen pressure from suction *P*_Su+_ as measured from the lip force measurements. Mean values from three measuements are used for all data points

It seems natural to investigate in what respect the new parameter *P*_Su+_ is related to the intraoral pressure that is present during suction. In Figure 5, the result from the pilot experiment is shown where the measured maximum negative differential pressure *P*_Vac_ is plotted against the OSP parameter *P*_Su+_. It was found that there seems to be a linear relation between *P*_Vac_ and *P*_Su+_, but in absolute values, the *P*_Vac_ is approximately twice the value of the OSP parameter *P*_Su+_.

## 4 DISCUSSION

### 4.1 Instruction

In this study, there is consistency between the values from Screens C and D and the values in a previous study where these screens had the most similar overall mean value (Wertsén & Stenberg, 2017). In the present study, clearer instructions have been used to help test persons to either squeeze or suck the oral screen. In Figure 5, it is shown that no values for semi‐individually made screens are found under the dotted line representing the same value on both the x‐axis and y‐axis. A majority of the values for the prefabricated screens are situated close to the line and under. This indicates that the test subjects easier could perform correctly according to the instructions using the semi‐individually made oral screens. The results for the instruction “Squeeze” show that the values of the A and B screens are relatively too high, reflecting that the test persons have failed to comply with the instruction and instead used suction. When giving the instruction “Suck,” there is little or no addition of pressure testing Screens A and B. In previous studies, prefabricated screens have been used, but no discussion has been made about how the possibility to mix “Suck” and “Squeeze” affects the measurement (Hägg & Tibbling, n.d.; Hägg et al., 2008; Sjögreen et al., 2011).

### 4.2 Oral screen design

The projected area for the soft oral screen decreased with 25% under load with the same forces that were measured with the testing subjects. This means that the active area is substantially smaller than the projected and the measured value of screen force might thus be smaller compared to a hard screen of similar size. This aspect has not been taken into account in studies performed with soft prefabricated oral screens (Sjögreen et al., 2007; Sjögreen et al., 2011).

When measuring lip force using prefabricated oral screens without a hole to let air in, it becomes difficult for the patient to execute the instruction “Squeeze.” However, when using a semi‐individual screen with a tube allowing air to pass, it is likely to get clearly separated values for the abilities squeezing and sucking. The screen design is obviously of great importance for the outcome. No studies have been found where oral screens with a similar design have been used.

### 4.3 Impact on test value

A problem when studying a normal population is that in healthy adults, the force when sucking is so high that the measuring procedure might be interrupted because of pain from the mucosa. This may result in a poorer performance in the first but mainly in the second session, the subjects having the experience of the first measurement in mind. In this study, the measured values for Screen D showed this phenomenon. Moreover, with Screen D, there was a great variation between the pressures performed by different healthy individuals compared to Screen C. This indicates some kind of impact on the measured values but no further analysis of this observation has been done in this study. However, a person with impaired oral motor function will probably never reach these high values.

### 4.4 Cut off value

To evaluate oral motor function in patients, it is desirable to have a cut off value for lip force that is independent of the equipment used. To make the value useful, it should be comparable to values from other studies. Therefore, a value of OSP expressed in kPa is preferable. In this study, we have proposed two independent parameters of oral screen pressure. One is related to the mechanical strength in the perioral muscles, *P*_Sq_, and one is also related to suction, *P*_Su+_. It is essential to have threshold values for both these parameters. A plot of longitudinal data in a two‐dimensional diagram such as Figure 5 could be a useful tool to diagnose and analyse therapeutic effects in patients. In other studies, values have been expressed in Newton, and no reflections have been made that values might differ with the size of the screen (Hägg & Tibbling, n.d.; Hägg et al., 2008; Sjögreen et al., 2007; Sjögreen et al., 2011).

### 4.5 Oral cavity pressure

The additional OSP as defined in this work is not identical to the vacuum in the oral cavity resulting from suction. One possible explanation could be how much tissue area actually is in contact with the screen during suction. The mechanism of how these parameters are related is not the subject of this work and should be interesting to study in future works.

### 4.6 Diagnostic possibilities

Santander et al. (2013) showed that the intraoral compartment pressures mainly are negative when swallowing (Santander et al., 2013). When examining patients with swallowing impairment, it is important to be able to diagnose if there is a problem creating negative intraoral pressure and/or to execute enough pressure in the perioral musculature. This study shows that when using a screen with a tube allowing air to pass, it is possible to get clearly separated values for the abilities squeezing and sucking. The significance of this opportunity has to be further investigated in future studies on patients with impaired oral motor function.

## 5 CONCLUSIONS

1. The instruction:
  - To suck generally increases the measured lip force.
  - The instruction is an important mean to get the correct measurements to distinguish between squeezing and sucking.
  - Measurements with the prefabricated screens indicate that the subjects mix squeezing and sucking in spite of the instructions.
2. The design of the oral screen:
  - The lip force increases in relation to the projected area of the screen.
  - It is possible to avoid suction when squeezing if the screen is designed with a tube allowing air to pass.
3. The additional screen pressure when sucking is related to the ability to produce a negative intraoral pressure.

## CONFLICT OF INTEREST

The authors declare that they have no conflict of interest.
